# Safety in Mixed Martial Arts: a 7-Year Review of Cancelled MMA Bouts in Calgary, Alberta, During the Pre-bout Medical Examination Period

**DOI:** 10.1186/s40798-018-0119-2

**Published:** 2018-01-12

**Authors:** Gwynn Curran-Sills

**Affiliations:** Family Medicine and Primary Care Research Office, University of Calgary, G012, Health Sciences Centre 3330 Hospital Drive NW, Calgary, Alberta T2N 4N1 Canada

**Keywords:** Mixed martial arts, Epidemiology, Injury, Head trauma

## Abstract

**Background:**

Presently, there is no literature that examines the reasons for the cancellation of amateur or professional mixed martial arts (MMA) bouts. The purpose of this study was to review the circumstances that lead to the cancellation of MMA bouts by Calgary ringside physicians during the pre-bout examination period and to identify any emerging patterns that may guide the regulatoin of this sport.

**Methods:**

The case-series  was constructed from the Calgary Combative Sports Commission pre-bout examination records and the medical records submitted by each athlete from January 2010 to December 2016.

**Results:**

Cancelled bouts in the pre-bout examination periods represented 5.4% of all MMA bouts in Calgary. A total of 25 reasons lead to bout cancellation and included the following: failure to obtain required neuroimaging (28.0%), neuroimaging abnormalities (24.0%), incomplete routine screening investigations (16.0%), exceeding maximum weight differential between the two athletes (16.0%), injury in the pre-competition period (8.0%), dehydration (4.0%), and ECG abnormalities (4.0%). The abnormalities on neuroimaging (*n* of 6) included the following: post traumatic gliosis on MRI (*n* = 1, 16.7%), flares diffusely and findings consistent with microhemorrhage on MRI (*n* = 1, 16.7%), chronic orbital fracture with fat pad extrusion on CT (*n* = 2, 33.3%), lacunar infarct on MRI (1), and unspecified MRI abnormality (*n* = 1, 16.7%). Twenty-two athletes had bouts cancelled and of these three athletes had their bouts stopped for two reasons.

**Conclusions:**

The following recommendations are presented and include: the creation of guidelines regarding pre- and post-bout neuroimaging, the implementation of industry-wide minimum medical screening standards, the adoption of a longitudinal approach to weight monitoring, the development of competent ringside physician groups, and active oversight by the Combative Sports Commission during the matchmaking process.

## Key Points

- Cancelled bouts in the pre-bout examination periods represented a small proportion (5.4%) of all MMA bouts in Calgary.
- Microstructural changes and bony abnormalities accounted for the majority of neuroimaging findings that lead to cancelled bouts.
- The creation of industry-wide minimum medical screening standards and the adoption of a longitudinal approach to weight monitoring should be considered by MMA regulatory bodies.

## Background

Mixed martial arts (MMA) is a relatively young sport for which there is a limited but growing body of literature that has focused on descriptive epidemiology of injuries obtained in training [1], descriptive epidemiology and risk factors associated with injuries sustained during competition [2–7], risk factors associated with head trauma during competition [8], the physiological and performance characteristics of successful athletes [9–11], and weight cutting practises employed by MMA athletes [12–15]. Working within this framework of knowledge, the American [16], Australian [17], British [18], and Canadian Medical Associations [19] have called for a ban of the sport based upon assumptions that it is unregulated, with minimal rules, and more violent and injury prone than other sports. Despite the reservations of these medical bodies, MMA continues to grow in spectatorship [20–22]. The call for a ban on the sport has not deterred the burgeoning interest and increasing athletic pursuit in the sport both on an amateur and professional level [23]. Given this escalation in the popularity of MMA, it is prudent for the medical community to use an evidence-based approach to identify areas of risk for MMA athletes. Once identified, the creation of interventions to reduce these risks can be adopted by sanctioning bodies to better protect these athletes.

For MMA in Canada, the regulatory body oversees athlete health considerations in the peri-competition period and regulation of professional and amateur MMA bouts falls under the authority of combat sport commissions across the country. Depending on the jurisdiction, a commission’s authority will either be associated with a municipality or a provincial government. Regulation in the USA [24] and Australia [25] is similar; however, for the UK, there is no formal regulatory body [12]. Similar governmental regulation exists for professional boxing and Muay Thai. However, amateur boxing and Muay Thai are generally not regulated by governmental bodies and instead are overseen by the associated Boxing and Muay Thai Amateur Associations.

An athlete can participate in a MMA bout when he/she obtains a licence from the appropriate commission. This process involves specific bureaucratic and medical requirements that vary between jurisdictions. At present, there is no literature that examines the rationale for why MMA bouts are cancelled during the pre-bout medical examination period. The focus for this study is to review the circumstances that lead to the cancellation of MMA bouts by Calgary ringside physicians during the pre-bout examination period and to identify any emerging patterns that may guide the regulation of this sport.

## Methods

This project was a secondary analysis of data collected for a retrospective cross-sectional study of Calgary Combative Sports Commission (CCSC) records from January 1, 2010 to December 31, 2016 in Calgary, Alberta, Canada. The records included both professional and amateur level athletes. Please see reference [26] for a detailed description of the primary study and dataset. In addition to the licencing requirements, the CCSC conducted pre- and post-bout medicals for each athlete that competed in the city of Calgary. Within Alberta, Canada, combative sports are not regulated at a provincial level, but instead at the municipal level. The pre- and post-bout medicals were performed by physicians with nursing support. The cohort of athletes who had their contests cancelled was constructed from the CCSC pre-bout examination records and the medical records submitted by each athlete. The pre-bout medical examination occurred the day before the event in conjunction with the CCSC weigh-in process. As part of the pre-bout medical examination, athletes submitted and partook in the following: clearance from their family physician to compete; a review of their past medical and surgical history, medications, and allergies; a complete physical examination with system-focused review as necessary; screening for infectious disease (Human Immunodeficiency Virus, Hepatitis B and C within the last 6 months); screening for pregnancy (less than 7 days prior the bout); screening ECG within the last year; a fully dilated funduscopic examination within the last year; and a screening magnetic resonance imaging (MRI) of the head for any athlete that previously competed in an unsanctioned bout. Athletes who did not fulfil the pre-bout medical requirements had their bouts cancelled and received a suspension as determined by the examining physician. This suspension information was recorded by the CCSC and forwarded to the Association of Boxing Commissions (ABC) to be disseminated to all the commissions that are part of the ABC. This step is taken to prevent the athlete from being able to compete in another sanctioned amateur or professional bout in a different jurisdiction.

Descriptive statistics were generated and where possible averages and standard deviations were calculated, along with the range. These calculations and tables were constructed using Microsoft Office Excel™ (Redmond, Washington, USA). Regular ringside physician coverage was defined as greater than 3 years of working with the CCSC or a minimum of 12 events worth of experience. An athlete’s bout experience was defined as the sum of all previous sanctioned contests. The CCSC defined a maximum weight differential between two athletes’ as 3.2 kg for Atomweight; 5.4 kg for Straw-, Fly-, Bantam-, Feather-, and Lightweight; 6.8 kg for Welter- and Middleweight; 9.1 kg for Light heavyweight; 13.6 kg for Heavyweight; and 18.1 kg for Super Heavyweight [27]. If this criterion was met, the bout was automatically cancelled. A physician clinically determined if an athlete was dehydrated (i.e., through mental status examination, vital signs, and clinical gestalt); however, there was no formal definition that was used by the ringside physicians who covered these events and this determination was subjective. There was no definition for an injury that resulted in cancellation of a bout; this was at the discretion of the ringside physician. The MMA promotional organization bout cancellation percentage was defined as the (number of cancelled bouts/number of hosted bouts) × 100.

## Results

From January 2010 to December 2016, 46 MMA events were held in Calgary and this generated 390 bouts (see reference [26] for a detailed description of that dataset). Over this time frame, 21 bouts (5.4%) were cancelled during the pre-bout examination period with 22 athletes having reasons that contributed to the bout cancellation. Table 1 outlines the event and athlete characteristics, along with the reason for why each athlete was not allowed to compete. Athletes involved in cancelled bouts had an average age of 29.7 (± 5.9, 21–39) and 86.4% were male. The average body mass was 81.3 kg (± 23.4, 52.1–120.2) (contracted weights were used for three athletes), with the average mass (where possible to calculate) as a function of weight class being 59.4 kg (± 2.6, 57.6–61.2) for bantamweight, 80.6 kg (± 6.2, 77.1–87.8) for welterweight, 85.0 kg (± 1.4, 83.9–85.7) for middleweight, 92.5 kg (± 0.6, 92.1–93.0) for light heavyweight, and 114.4 kg (± 7.0, 103.4–120.2) for heavyweight. Of the 21 cancelled bouts, 71.4% were scheduled professional contests. Athletes with cancelled bouts where predominantly from Canada (72.7%), but also recorded country of origin as the USA (18.2%), Brazil (4.5%), and Peru (4.5%). The average athlete bout experience was 8.5 bouts (± 12.3, 0–44), with 18.2% of athletes having no prior sanctioned MMA experience. Cancelled bouts came from 11 events that were run by four different MMA promotional organizations.

**Table 1:** Medical reasons and characteristics of athletes involved in cancelled bouts in Calgary, Alberta, from January 2010–December 2016

Athlete	Event name^*^ and year it occurred	Competition level (Rounds × Time)	Province, country	Body mass (kg)	Sex and age (years)	Record (W-L-D)	Reason for cancellation of the bout	Physician^^^
1	A1 2010	Pro (3 × 5)	Alberta, Canada	86.0	M 25	P:1-1-0, A: 0-0-0	The bout was cancelled the night of the event due to a knee injury sustained by the athlete earlier that day. The athlete self-referred to the ringside physician and the athlete was placed on indefinite suspension until further clinical assessment and imaging was completed.	X1
2	A1 2010	Amt (3 × 3)	Alberta, Canada	87.8	M 21	A: 0-0-0	Exceeded maximum weight differential (> 6.8 kg) between athletes and CCSC cancelled the bout.	X1
3	A2 2011	Amt (3 × 3)	Alberta, Canada	57.6	M 21	A: 0-0-0	Did not complete routine screening blood work.	X2, Y1
4	B 2011	Pro (3 × 5)	Alberta, Canada	117.5	M 32	P:3-5-0, A: 0-0-0	Unspecified MRI (head) abnormality. Indefinite suspension until neurologist clearance. Unclear why the MRI was obtained.	X3, Y2
5	C 2012	Pro (3 × 5)	Alberta, Canada	66.5	M 28	P:1-2-0, A: 0-0-0	Missed weight and clinically dehydrated (abnormal vital signs). Started oral rehydration and transported for IV hydration. Indefinite suspension and required to demonstrate normal kidney function prior to next bout.	Y2, Y3
6	A3 2014	Amt (3 × 3)	Ontario, Canada	83.9	M 28	A: 2-0-0	MRI (head) revealed post traumatic gliosis. Indefinite suspension until cleared by neurologist. MRI required by another commission post KO/TKO.	Y4, Y5
7	D1 2015	Pro (3 × 5)	Alaska, USA	77.1	M 25	P:12-4-1, A: 0-0-0	Did not obtain a diluted fundoscopic exam or MRI (head) for NSF.	Y2, Y4
8	D1 2015	Pro (3 × 5)	Alberta, Canada	93.0	M 37	P:0-1-0, A: 5-0-0	Athlete sustained unspecified injury the day of the weigh-in and declined to compete after review with the ringside physician.	Y2, Y4
9	D1 2015	Pro (3 × 5)	British Columbia, Canada	56.5	M 36	P: 0-0-0, A: 0-0-0	Did not obtain an MRI (head) for NSF.	Y2, Y4
10	A4 2015	Amt (3 × 3)	Alberta, Canada	& (N/A)	M 23	A: 1-1-0	Did not obtain an MRI (head) for NSF.	Y3, Y4, Y6
11	A4 2015	Pro (3 × 5)	Ontario, Canada	& (52.1)	F 33	P: 2-1-0, A: none	Did not obtain MRI (head) for NSF. Did not produce prior CT (head), this study was completed by another commission.	Y3, Y4, Y6
12	A4 2015	Pro (3 x 5)	Lima, Peru	& (52.1)	F 28	P: 2-1-0, A: 0-1-0	Did not obtain an MRI (head) for NSF.	Y3, Y4, Y6
13	A4 2015	Pro (3 × 5)	Ontario, Canada	& (83.9)	M 34	P: 1-2-0, A: none	Exceeded maximum weight differential (> 6.8 kg) between athletes and CCSC cancelled the bout.	Y3, Y4, Y6
14	D2 2016	Pro (3 × 5)	Washington, USA	77.1	M 28	P: 15-8-0, A: 1-1-0	Did not obtain an MRI (head) for NSF.	Y5
15	D2 2016	Pro (3 × 5)	British Columbia, Canada	54.4	F 36	P: 2-4-0, A: 4-1-0	MRI (head) for NSF showed flares diffusely and finding consistent with microhemrrhage. Indefinite suspension until neurologist clearance.	Y5
16	D2 2016	Amt (3 × 3)	Ontario, Canada	111.1	M 22	A: 0-0-0	Did not obtain an MRI (head) for NSF.	Y5
17	D2 2016	Amt (3 × 3)	Ontario, Canada	54.4	F 26	A: 0-0-0	Did not complete routine screening blood work.	Y5
18	A5 2016	Pro (3 × 5)	Sao Paulo, Brazil	103.6	M 37	P: 1-2-0, A: 0-0-0	CT (head) for NSF showed chronic orbital fracture with fat pad extrusion. Indefinite suspension until clearance by Maxillary Facial surgeon. Imaging obtained as requirement of another commission.	Y2, Y3, Y6
19	A5 2016	Pro (3 × 5)	Alberta, Canada	92.1	M 39	P:20-24-0, A: 0-0-0	Cancelled due to lack of anticonvulsant levels within the last year.	Y2, Y3, Y6
20	A6 2016	Pro (3 × 5)	Manitoba, Canada	61.2	M 24	P: 3-0-0, A: 3-0-0	Significant weight difference (> 5.5 kg) between athletes and CCSC cancelled the bout.	X4, Y2, Y3
21	A7 2016	Pro (3 × 5)	North Carolina, USA	120.2	M 35	P:26-12-1, A: 0-0-0	CT (head) for NSF showed chronic orbital fracture with fat pad extrusion. Indefinite suspension until clearance by Maxillary Facial surgeon. Imaging obtained as requirement of another commission.	Y3, Y5, Y6
22	A7 2016	Pro (3 × 5)	Michigan, USA	119.3	M 36	P: 6-2-0, A: 2-0-0	MRI (head) for NSF showed lacunar infarcts. Elevated blood pressure. ECG biphasic t-waves in V4, T-wave inversion V5-6 and all inferior leads. Indefinite suspension until cardiology consultation.	Y3, Y5, Y6

*NSF* non-sanctioned fight, *N/A* not available, *P* professional record, *A* amateur record, *ECG* electrocardiogram

*The letter represents a distinct MMA promotional organization and the number denotes multiple events put on by this organization

^**^**^The X and Y represent physicians that do not and those that do provide regular coverage as a ring-side physician, respectively. The number represents a unique physician

^&^Did not weigh-in prior to bout being cancelled. The weight provided in the bracket represents the agreed contract weight for the bout

Of the 390 bouts held in Calgary, over the study period, 371 were run by organizations that had cancelled bouts in the pre-bout examination period (Table 2) and the remaining bouts were hosted by two promotional organizations that had no cancelled bouts. The number of bouts and the number of cancelled bouts as a function of MMA promotional organization are represented in Table 2. Over the study period, 5.4% of all bouts were cancelled. The average MMA promotional organization bout cancellation percentage was 13.2% (± 24.9, 0–63.6). However, the bout cancellation percentage for all organizations were similar exception for promotional organization D, which showed a bout cancellation percentage of 63.6%.

**Table 2:** Number of cancelled bouts and cancellation rate for MMA promotional organizations in Calgary, Alberta from January 2010–December 2016

MMA Promotional Organization	Number of bouts (%)	Number of cancelled bouts (%)	Bout cancellation percentage
A	326 (83.6)	12 (57.1)	3.7
B	15 (3.8)	1 (4.8)	6.7
C	19 (4.9)	1 (4.8)	5.3
D	11 (2.8)	7 (33.3)	63.6
E	11 (2.8)	0 (0)	0.0
F	8 (2.1)	0 (0)	0.0
Total	390	21	N/A

Twenty-two athletes had bouts cancelled during the pre-bout medical examination period, with three athletes having two reasons for cancelling their bout. A total of 25 reasons lead to bout cancellations and included the following: failure to obtain required neuroimaging (*n* = 7, 28.0%), neuroimaging abnormalities (*n* = 6, 24.0%), incomplete routine screening investigations (*n* = 4, 16.0%), exceeding the maximum weight differential between the two athletes (*n* = 4, 16.0%), injury in the pre-competition period (*n* = 2, 8.0%), dehydration (*n* = 1, 4.0%), and ECG abnormalities (*n* = 1, 4.0%). The abnormalities on neuroimaging (n of 6) included the following: post traumatic gliosis on MRI (*n* = 1, 16.7%), flares diffusely and findings consistent with microhemorrhage on MRI (*n* = 1, 16.7%), chronic orbital fracture with fat pad extrusion on CT (*n* = 2, 33.3%), lacunar infarct on MRI (*n* = 1, 16.7%), and unspecified MRI abnormality (*n* = 1, 16.7%).

The number of ringside physicians at an event was at least one, as mandated by the CCSC [27], but varied from one to three physicians depending on size of the event being run by the MMA promotional organizations. Ten physicians were involved in the events in which a contest was cancelled in the pre-bout medical examination period. The clear majority of the events with cancelled bouts (90.9%) were covered by six regular ringside physicians.

## Discussion

This is the first study to provide perspective on medical and non-medical reasons for the cancellation of MMA contests during the pre-bout examination period conducted by an athletic commission. While the descriptive epidemiology is relatively simple, and further investigation of this area is required, it does highlight several areas that require attention.

### Abnormalities on Imaging of the Head

The frequency of neuroimaging or incidence of abnormality on neuroimaging in this cohort of athletes that competed in Calgary from January 2010 to December 2016 is not possible to extrapolate. This occurred because the baseline number of athletes that required any neuroimaging (as an example, imaging in the post bout period) could not be determined from CCSC records over the entire study period (records were only maintained for 1 year period before destruction prior to 2014). What can be inferred is that 780 athletes competed over the 7-year study period, and 5 were identified with abnormal neuroimaging that lead to an indefinite suspension in the pre-bout period. Apart from the lacunar infarct finding (Athlete 22 in Table 1 was also a poorly controlled hypertensive patient despite medication, along with electrocardiogram abnormalities), each of the other neuroimaging abnormalities is in keeping with a traumatic head mechanism of injury [28–30]. More specifically, there is emerging evidence from MMA and boxing literature suggesting that the microstructural changes observed on these investigations could be related to previously sustained training or competition head trauma [31, 32] and potentially correlate with chronic traumatic encephaolopathy [33]. This is especially concerning as Hutchinson et al. reported a knock out rate of 6.4 per 100 athlete-exposures for MMA athletes, and those who were knocked out on average sustain 2.6 head strikes after they have lost consciousness prior to referee intervention [8].

The ABC Medical Committee provides minimal guidance on the use of neuroimaging in the pre-bout examination period and no guidance on the use of neuroimaging for post-bout examination [34]. Presently, this is a nebulous area for ringside physicians where there are many more questions than definitive answers. Overarching themes include the following: What is the frequency of screening neuroimaging and does this depend of the athlete’s age? Is neuroimaging warranted after an unsanctioned bout? What are the needs for neuroimaging post traumatic brain injury? What is the appropriate type of neuroimaging? While a thorough review of the literature relating to neuroimaging of head trauma from a combative sport perspective is beyond the scope of this manuscript, the approach to these scenarios would benefit from a consensus or guidelines statement from the ABC Medical Committee or the Association of Ringside Physicians (ARP).

### Minimum Medical Screening Standards

The minimum medical screening standards (MMSS) are vitally important as they set a benchmark for what is deemed medically necessary to allow an athlete to compete [24]. The CCSC has used screening medical requirements that are in keeping with the precedent-setting sanctioning bodies in the USA such as Nevada or New Jersey State Commissions [35–37]. As such, the CCSC has incorporated screening for infectious blood borne pathogens, pregnancy, and cardiac, ophthalmological, or neurological disease.

Presently, there is no uniformity between commissions across Canada, let alone from the international context, when it comes to MMSS. Review of the MMSS across the members on the ABC website will quickly highlight the wide variations between commissions [24]. This can range from conducting a pre-bout physical the day of the event to a pre-bout physical plus screening for infectious blood borne pathogens, pregnancy, and cardiac, ophthalmological, or neurological disease and additional requirements for older athletes. The ABC Medical Committee has outlined MMSS [34] for combative sports athletes. However, these are merely suggestions and do not have to be incorporated into a commission’s standard practice for that commission to maintain membership in the ABC. In an effort to create uniformity, the ABC and the ABC Medical Committee should institute MMSS that must be followed by a commission if it is to be entitled to membership in the ABC.

### Weight Cutting and Dehydration

The policy around weight cutting is an active area of review for regulatory bodies [38] with no uniformly accepted approach. In this data set, only a single athlete (Table 1, Athlete 5) was suspended from competition secondary to dehydration in the pre-bout examination period. Surprisingly, this indicates that 0.13% of MMA athletes that reported to the CCSC weigh-in were considered dehydrated. However, there was no formal definition used to determine if athletes were dehydrated or a means to estimate the level of dehydration. As such, it is not possible to infer if this data set is representative of the number of athletes that are dehydrated when they present for weigh-in. There is no literature to support this negligible number of athletes showing evidence of dehydration, instead, there is mounting literature identifying the trends of rapid weight cutting practice in MMA [12, 13, 39] and other combative sports [14]. This literature supports that dehydration has become a normal part of the weight-cutting culture in MMA [12, 13]. A recent investigation by Matthews et al. [13] studied MMA athletes’ weight-making practices and discovered that at the official weigh-in, 57% of athletes were dehydrated and the remaining 43% were severely dehydrated according to their urinary hydration status. Jetton et al. [39] identified that 39% of MMA athletes remained significantly or seriously dehydration 2 h prior to competition despite the official weigh-in process having occurred 22 h prior. Dehydration in combative sports has been linked to tangible health consequences. For example, among other risks, it can leave athletes susceptible to closed-head trauma [40] and transient cognitive impairment [41] when athletes are still dehydrated.

Over the last year and a half, a new approach has been adopted by some American commissions that allows for an early weigh-in process [42, 43] to give athletes more time to rehydrate before the commencement of the bout in an effort to decrease the aforementioned dehydration risks. Specifically, this new weigh-in procedure offers athletes several more hours than the customary 24 h prior to competition in which to rehydrate. The California State Athlete Commission in May 2017 passed a 10-point weight-cutting regulation [38] that endorsed the extended rehydration period and proposed several recommendations to curb extreme weight cutting. Alternatively, One Championship has implemented a much more progressive approach to prevent extreme weight cutting [44]. An athlete’s competing weight class is assigned by One Championship based upon their current walking weight and daily training weights. Once the competition weight class has been established, the athlete cannot alter their weight class fewer than 8 weeks prior to the event and One Championship can conduct random weight checks leading up to the event. These two approaches highlight very different interventions—one that tries to reverse the effects of extreme weight cutting and the other that tries to prevent extreme weight cutting from occurring. Intuitively, the longitudinal approach offers a means to reframe the weigh-in process and mitigate extreme weight-cutting practices. However, the adoption of such a process will need to overcome imbedded weight-cutting practices in MMA culture and will require consistent support from the sanctioning and promotional organizations in which the athletes are competing. Beyond the initiation of any new weigh-in practice, there should be further efforts to scientifically validate the need for such measures and subsequent investigation to show that the new practice is creating a healthier or safer process of the athlete.

### Support for Athletes to Withdraw from Competition

According to the data, two athletes (Table 1, Athletes 1 and 8) withdrew from competition of their own volition secondary to injuries. In each of these incidences, the athletes sought the withdrawal from competition after conferring with the ringside physician. Partaking in amateur or professional MMA bouts creates not only an internal spirit of competition in the athlete but also external expectations from their coaches, the promotional organization, and the fans [45]. Having a medical team that is not affiliated with any of these groups provides credibility and support for the athlete’s health concerns and creates a space where the athlete can make an informed decision regarding competition [46, 47].

### Developing Competent Ringside Physicians

Establishing a competent group of ringside physicians with a consistent approach to peri-competition medical screening and suspensions is essential for athlete safety. As there continues to be wide variation in local practice by commissions [24] when it comes to screening for head-related trauma, MMSS, and weigh-in procedures, ringside physician groups need to work collaboratively to aid with mentoring any new physician that joins the group. The creation of a competent group of ringside physicians that understands not only the medical aspects of care but also the regulatory, social, and economic forces within MMA is necessary when balancing athlete safety. To further develop ringside physician competency, the ARP, along with the American Colleague of Sports Medicine, provides the Certified Ringside Physicians program [48]. In addition, the ARP offers many online resources to assist physicians with peri-competition medical screening and health challenges, as well as continuing medical education [49].

### Training and Bout Cancellation

Injury disclosure by an athlete (Table 1, Athletes 1 and 8) that prevented them from competing in sanctioned contests in Calgary from January 2010 to December 2016 accounted for 8% of cancelled bouts or 36.6 injuries per 100,000 athlete-years (calculated as [2/(780 × 7)] × 100,000). It is not possible to ascertain from the data if these injuries were related to training or another mechanism of injury. Even if it is assumed that these two injuries occurred during training, this is a low occurrence of training-related injuries. A single study from the MMA literature reported training-related injuries as 376.4 per 100 athletes (the time frame for this study could not be determined) [1]. The literature on training-related injuries in other combative sports shows the following: for boxing, it is reported as 16.2 to 19.2 per 100 athlete-year [50, 51]; for karate, its ranges from 20.2 per 100 athletes (the time frame for this study could not be determined) [52] to 45.2 per 100 athlete-year [53]; for taekwondo, it is reported as 7.1 to 92.8  per 100 athlete-year [50, 54]; for judo, it ranges from 8.2 to 29.6 per 100 athlete-years [50, 55]; and for wrestling 132.0 per athlete-years [56] has been found. Additionally, there is a spectrum when it comes to the proportion of injuries occurring in the training phase of different combative sports: for boxing, it is 5.3 to 42.9% [50, 51]; karate, it is 75.9% [53]; taekwondo, it is 36.0 to 81.5% [50, 54]; judo, it is 11.6 to 70.0 [50, 56]; and wrestling, it is 63.0% [56]. It is not clear why there is such a large discrepancy between the existing literature and the rate of presumed training-related injuries in this study. However, this may be explained by any of the following reasons: bouts were cancelled due to athlete injury by the promoter before the pre-bout examination period; athletes were not volunteering the existence of injuries; the screening techniques employed by ringside physicians did not detect the injuries; or the injuries were considered minor by the ringside physician and medical clearance was given. Ultimately, additional study of the pre-competition period is necessary to better quantify the existence and frequency of injury during this phase of a MMA athlete’s career.

### Matchmaking and Combative Sports Commission Oversight

Cancelled bouts in the pre-bout examination periods represented 5.4% of all MMA bouts in Calgary over the study period. When looked at from a MMA promotional organization standpoint, the average bout cancellation percentage is 13.2% (± 24.9, 0–63.6). However, promotional organization D (Table 2) appears to be an outlier at 63.6%, and if this is removed, the average bout cancellation percentage drops to 3.1% (± 3.0, 0–6.7). There is presently no combative sports literature that reports on the occurrence of cancellations in the pre-bout period for comparison.

What is highlighted by this finding is a consistent bout cancellation percentage for all organizations but one. This large discrepancy for promotional organization D could be related to the following: the small number of events and bouts held by the organization, limited experience with the matchmaking process, or its ability to attract seasoned amateur and professional athletes may have been limited as it was a younger organization in Calgary. However, promotional organizations B, C, E, and F would also be considered young organizations in Calgary and did not suffer from the same large bout cancellation percentage. The CCSC is responsible for licencing promotional organizations, along with the athletes, and approving the matchmaking process. As such, it behoves the commission to guide new or younger promotional organizations through the matchmaking process to ensure that athletes are safely chosen to engage in competition prior to the pre-bout medical examination. Alternatively, if there is an emerging pattern of athlete safety concerns, as noted by cancelled bouts, then the commission should consider not granting a licence to such a promotional organization.

## Limitations

The matchmaking process is extremely dynamic, and as such, there tends to be bouts that do not materialise for a host of reasons. Most of these cancelled bouts will not come to the attention of the CCSC as they will not make it to the pre-bout period in which the commission is involved. Quantifying this number of cancelled bouts in the matchmaking process or the reasons for such is not possible through the commission records but should be considered for future study. There were no means to ascertain if all of the contests cancelled by the CCSC in the pre-bout examination period were captured in this data set as the commission only maintained their records for 1 year after the event prior to 2014.

The clear majority of contests that were cancelled were done so for reasons that did not require interpretation, i.e. failure to obtain required neuroimaging, neuroimaging abnormalities, incomplete routine screening investigations, exceeding the maximum weight differential between the two athletes, and ECG abnormalities. However, there was subjectivity when it came to physician interpretation of the degree of pre-existing athlete injury, the presence of dehydration, or the level of dehydration that lead to the bout being cancelled. It was not possible to quantify how clinical gestalt may have affected the number of bouts that were cancelled or allowed to continue when injury or dehydration was present. Future work could consider implementing a priori definitions for degree of injury or dehydration to create more consistency when determining if a bout is to be cancelled.

## Conclusions

A relatively small number of amateur and professional MMA bouts were cancelled during the pre-bout medical examination period in Calgary, Alberta, from January 2010 to December 2016. The reasons for bout cancellation included the following: failure to obtain required neuroimaging, neuroimaging abnormalities, the athlete did not complete the routine screening investigations, exceeding the maximum weight differential between the two athletes, injury in the pre-competition period, dehydration, and ECG abnormalities. The following recommendations are presented and include the creation of guidelines regarding pre- and post-bout neuroimaging, the implementation of industry-wide minimum medical screening standards, the adoption of a longitudinal approach to weight monitoring, the development of competent ringside physician groups, and the active Combative Sports Commission oversight during the matchmaking process.

## Abbreviations

ABC: Association of Boxing Commissions
ARP: Association of Ringside Physicians
CCSC: Calgary Combative Sports Commission
ECG: Electrocardiogram
MMA: Mixed martial arts
MMSS: Minimal medical screening standards
MRI: Magnetic resonance imaging
